# Trends in Opioid Medication Adherence During the COVID-19 Pandemic: Retrospective Cohort Study

**DOI:** 10.2196/42495

**Published:** 2023-09-01

**Authors:** Amir Marashi, David Warren, Gary Call, Mark Dras

**Affiliations:** 1 School of Computing Macquarie University Macquarie Park Australia; 2 Gainwell Technologies Tysons, VA United States

**Keywords:** COVID-19, opioid crisis, opioids, medication for opioid use disorder, MOUD, pandemic, public health, opioid, medication, treatment, care, patient, opioid use disorder, beta regression analysis, breakpoint analysis

## Abstract

**Background:**

The recent pandemic had the potential to worsen the opioid crisis through multiple effects on patients’ lives, such as the disruption of care. In particular, good levels of adherence with respect to medication for opioid use disorder (MOUD), recognized as being important for positive outcomes, may be disrupted.

**Objective:**

This study aimed to investigate whether patients on MOUD experienced a drop in medication adherence during the recent COVID-19 pandemic.

**Methods:**

This retrospective cohort study used Medicaid claims data from 6 US states from 2018 until the start of 2021. We compared medication adherence for people on MOUD before and after the beginning of the COVID-19 pandemic in March 2020. Our main measure was the proportion of days covered (PDC), a score that measures patients’ adherence to their MOUD. We carried out a breakpoint analysis on PDC, followed by a patient-level beta regression analysis with PDC as the dependent variable while controlling for a set of covariates.

**Results:**

A total of 79,991 PDC scores were calculated for 37,604 patients (age: mean 37.6, SD 9.8 years; sex: n=17,825, 47.4% female) between 2018 and 2021. The coefficient for the effect of COVID-19 on PDC score was –0.076 and was statistically significant (odds ratio 0.925, 95% CI 0.90-0.94).

**Conclusions:**

The COVID-19 pandemic was negatively associated with patients’ adherence to their medication, which had declined since the beginning of the pandemic.

## Introduction

The opioid crisis is an ongoing problem in the United States. Recent reports and studies have shown an increase in the number of overdoses in 2020, suggesting an association between the COVID-19 pandemic and the worsening of the opioid crisis [1-3]. Disruptions in medical services and care due to lockdowns, stay-at-home orders [4], and the scarcity of health care services might be some of the factors involved in this association. Patient adherence to medications is an important factor in the success of medication for opioid use disorder (MOUD), and higher adherence is associated with a reduction in mortality rate [5]. Pharmacists, health care experts, and public and private health technology companies have expressed their concerns about medication adherence amid the pandemic, both in the United States [6,7] and internationally [8-11]. Increases in emergency medical services responses, including the administration of naloxone [12], suggest that these concerns are well founded.

A recent study [13] examined the changes in the prescribing of opioid analgesics and buprenorphine for opioid use disorder (OUD) throughout the COVID-19 pandemic. By looking at aggregate prescription levels, it found that existing patients who receive opioid analgesics and buprenorphine for OUD generally maintained access to these medications during the COVID-19 pandemic, whereas the initiation of buprenorphine remained at a low rate through August 2020. Another study investigating patients’ access to chronic medications during the COVID-19 pandemic used a dichotomous measurement for patients’ adherence; it found that since the beginning of the pandemic, patients were less likely to discontinue OUD therapy (buprenorphine or naloxone) [14]. However, both aggregate analyses and studies similar to Clement et al [14] that use dichotomous variables can conceal more fine-grained, individual-level changes.

In this study, we examined the trend of medication adherence for US Medicaid patients with an OUD diagnosis from 6 US states who were on opioid medications from January 2018 to March 2021. We calculated the proportion of days covered (PDC) as a method of measuring medication adherence for each patient in the 3 months following the first visit for an opioid-related disorder or opioid dependence indication, according to International Classification of Diseases, 10th revision (ICD-10) codes, and calculated the average monthly PDC score in each month. Then, we examined the adherence trend across time and investigated the association between the pandemic and PDC score, as well as the association between the pandemic and opioid overdose in a 3-month time window.

## Methods

### Overview

We analyzed US Medicaid claims data for 6 US states. The data set is provided by HMS (a Gainwell Technologies company) through the Digital Health Cooperative Research Centre and comprises Medicaid claims (inpatient, outpatient, dental, and pharmaceutical), a few demographic characteristics of the patients (age, sex, zip code, and Medicaid enrollment periods), and their unique identifiers. The data set links their claims across time, different settings of care, and multiple episodes of enrollment. Diagnosis codes associated with the patient, procedures codes, prescription date, dosage, duration, and other prescription details are among the information available in the data set. The data set covers claims from 2015 to 2021 and comprises over 1.16 billion claims for over 14.2 million unique individuals.

We used a continuous measurement of adherence called PDC as a proxy for medication adherence. We defined PDC as the ratio of the number of days a patient could have their opioid treatment medication to the number of days in the period of interest.

We created a subset of patients with at least one relevant F11 diagnosis ICD-10 code (codes F11.1 opioid abuse; F11.2 opioid dependence; and F11.9 opioid use, unspecified), who have filled their opioid treatment prescriptions in the 3-month window following the first visit with an F11 diagnosis code. We excluded the patients whose first F11 visit happened before 2018. For each patient with at least three months of opioid treatment medication, we calculated and assigned 3 monthly PDC scores for each month following their medical visit. To investigate the association between the COVID-19 pandemic and medication adherence, we took 2 separate approaches.

First, in an exploratory analysis, we plotted the average PDC score for all patients in each month to visualize any changes in the trend of the average PDC score since the beginning of the pandemic. To detect whether such changes exist and to avoid subjective judgements, we used an algorithm described by Bai and Perron [15] for simultaneous estimation of multiple breakpoints implemented in the *strucchange* R package [16]. To define how many breakpoints should be considered, we calculated the Bayesian information criterion (BIC) estimates for different numbers of breakpoints and chose the number that minimizes the BIC. The algorithm detects the time(s) where possible breakpoints occurred and the CIs for the detected times.

In the second approach, we applied a patient-level beta regression model to examine the effect of the COVID-19 pandemic (modelled as a binary variable with a value equal to 1 for months after March 2020) on the PDC score. We controlled for a set of other covariates, including the age, sex, state, and comorbidities of each patient. To reduce the dimensionality of comorbidities, we categorized diagnosis codes into Elixhauser general comorbidity categories [17] according to the Agency for Health Research Quality rules using the *icd* R package by Wasey and Lang [18]. The Elixhauser categories condense many dozens of ICD-10 (and other) administrative codes into 30 broader groupings (as termed by Elixhauser et al [17]), such as “Alcohol abuse” (ICD-10 codes F10, E52, etc) or “Drug abuse” (ICD-10 codes F11.x-F16.x, etc). In a separate model, we repeated the analysis with data inclusive of the patients with only 1 or 2 months of PDC scores to see the sensitivity of the model.

### Ethical Considerations

The data were provided in deidentified form by HMS. On this basis, an ethical review by Macquarie University deemed it as having negligible risk, in line with Australia’s National Statement on Ethical Conduct in Human Research 2007 (as updated in 2018).

## Results

**Figure 1:**
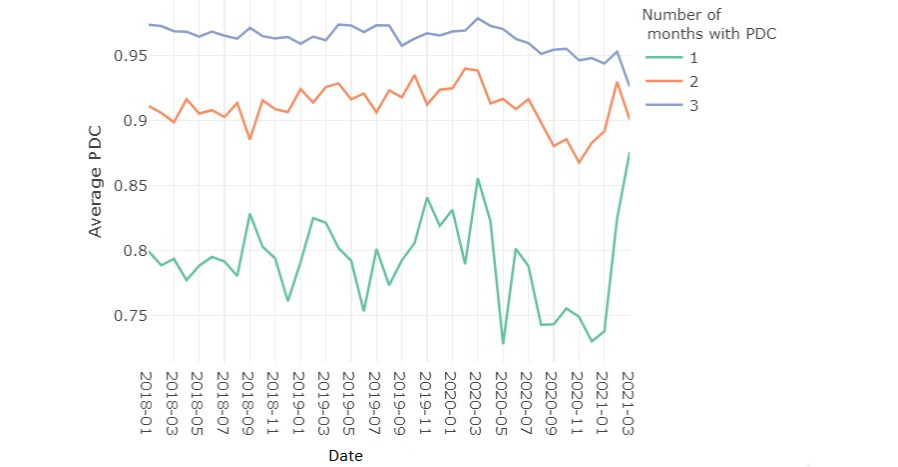
Average proportion of days covered (PDC) over time, a comparison of patients with 1, 2, and 3 months of PDC scores.

We calculated a total of 79,991 PDC scores for 37,604 patients in our subset OUD sample (age: mean 37.6, SD 9.8 years; sex: n=17,825, 47.4% female) between 2018 and 2021 from Medicaid claims data of 6 US states. Overall, 7816 patients who did not have a PDC score in the month following their F11 diagnosis or had a month without a PDC score between their first and second scores were excluded from the analysis. From the remaining patients, the most common treatment approach involved buprenorphine prescription (24,731/29,788, 83.02%), followed by prescriptions for naltrexone alone (3707/29,788, 12.44%); only small proportions were prescribed buprenorphine and naltrexone together (778/,29,788, 2.61%) or methadone (572/29,788, 1.92%). In terms of prescription filling patterns, only 24.9% (7417/29,788) filled their MOUD prescriptions in the month following their F11 diagnosis (mean PDC score 79.6), another 24.9% (7417/29,788) filled 2 months of MOUD prescriptions (mean PDC score 91.7), and 50.2% (14,954/29,788) filled 3 or more months of MOUD prescriptions (mean PDC score 96.1). To understand whether any drop in adherence as measured by PDC was related to patients discontinuing versus continuing OUD therapy, we separated out patients based on how many months were covered. As Figure 1 shows, the PDC score was lower for patients with fewer covered months. To focus on the change during the COVID-19 period for patients that were more adherent to their medication, for the main part of the analysis, we excluded the patients with less than 3 months of PDC scores.

**Figure 2:**
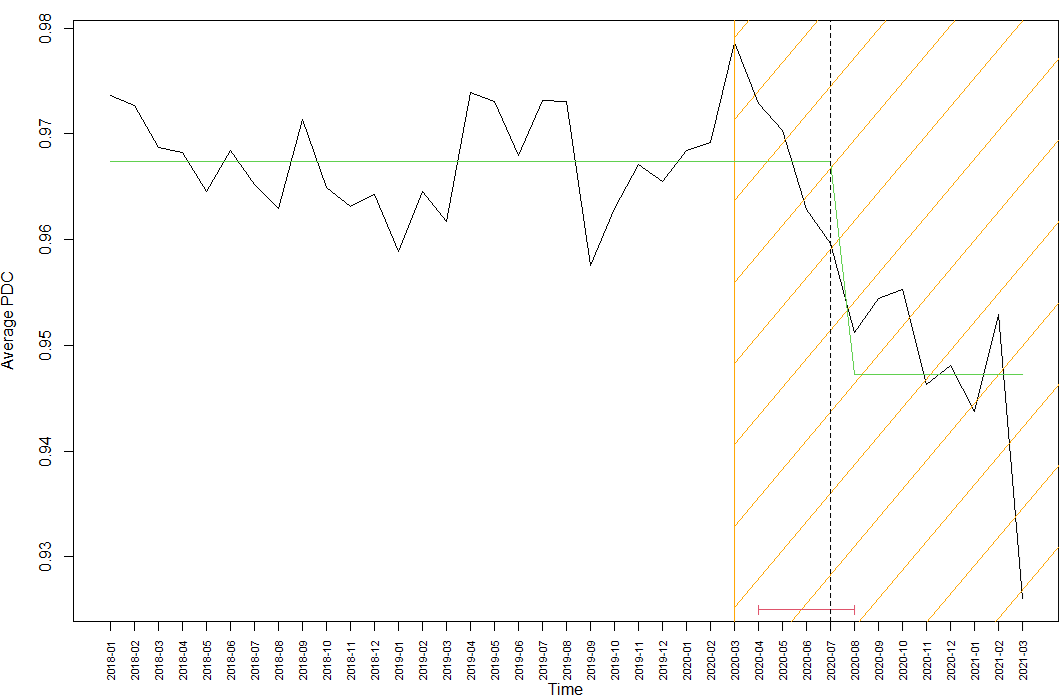
Detected breakpoints for patients with 3 months of proportion of days covered (PDC) scores.

Figure 2 shows the result of applying the breakpoint detection algorithm on the time series of the PDC scores for the selected patients. The shaded area denotes the time after the pandemic was announced (March 2020). The dash line represents the detected breakpoint by the algorithm in July 2020, and the red line represents the CI from April to August.

The results of the beta regression analysis with a logit link for features with statistically significant coefficients are reported in Table 1. The dependent variable is the calculated PDC score between 0 and 1 for each patient in each month. The independent variable of interest, named “COVID,” is a binary variable that is 0 for PDC scores at months before March 2020 and 1 for PDC scores from March 2020 onward. The control variables in the model included age group, sex, the month from which the PDC is calculated (PDC first month, PDC second month, and PDC third month), state identifiers, and Elixhauser comorbidity groups calculated based on the clinical history of each patient. Although the independent variables in the model did not explain much of the variance of the PDC score (*R*^2^ the model is 0.055), the coefficient for the effect of “COVID” was –0.76 and was statistically significant (odds ratio 0.925, 95% CI 0.90-0.94). This result is in line with findings of the exploratory analysis and breakpoint detection. The model inclusive of the patients with only 1 or 2 PDC scores showed similar results. The results showed that male patients had lower PDC scores (OR 0.97, 95% CI 0.96-0.99), whereas patients in all age groups older than the reference category (aged 17-29 years) had higher PDC scores. A history of obesity and alcohol use disorder were positively associated with the PDC score.

**Table 1:** The coefficients of the beta regressions models.

Feature name		Dependent variable: PDC^a^ score			
		Patients with at least 3 months of PDC scores		All patients	
		Value	*P* value	Value	*P* value
COVID, beta (SE)		–0.076 (0.010)	<.001	–0.081 (0.009)	<.001
**Age group (years), beta (SE)**					
	<16	–0.012 (0.262)	.96	0.173 (0.151)	.25
	30-39	0.027 (0.012)	.02	0.032 (0.010)	.002
	40-49	0.044 (0.014)	.002	0.041 (0.012)	.001
	50-59	0.045 (0.018)	.01	0.055 (0.016)	.001
	≥60	0.068 (0.031)	.03	0.084 (0.027)	.002
Sex, male, beta (SE)		–0.023 (0.009)	.01	–0.026 (0.008)	.002
**PDC month, beta (SE)**					
	Second	0.017 (0.011)	.12	0.022 (0.009)	.02
	Third	–0.018 (0.011)	.10	–0.025 (0.011)	.02
Number of months of PDC, beta (SE)		N/A^b^	N/A	0.254 (0.006)	<.001
Number of F11 codes, beta (SE)		–0.0001 (0.000)	.001	–0.0001 (0.000)	.001
**State, beta (SE)**					
	2	–0.041 (0.045)	.36	–0.051 (0.039)	.19
	3	–0.056 (0.022)	.01	–0.108 (0.019)	.001
	4	–0.084 (0.020)	.001	–0.124 (0.017)	<.001
	5	–0.036 (0.025)	.14	–0.052 (0.020)	.01
	6	–0.210 (0.013)	<.001	–0.308 (0.011)	<.001
**Elixhauser category names [17] for the relevant variables** **, beta (SE)**					
	Neuro other^c^	–0.038 (0.017)	.03	–0.015 (0.015)	.30
	Obesity	0.019 (0.017)	.25	0.030 (0.015)	.04
	FluidsLytes^d^	–0.034 (0.018)	.06	–0.022 (0.015)	.16
	Alcohol^e^	0.030 (0.018)	.09	0.098 (0.015)	<.001
	Drugs^f^	–0.011 (0.012)	.38	–0.020 (0.011)	.06
Constant		3.203 (0.027)	<.001	1.976 (0.027)	<.001
Observations, n		50,265	N/A	71,120	N/A
*R^2^*		0.055	N/A	0.137	N/A
Log likelihood		176,084.60	N/A	225,324.60	N/A

^a^PDC: proportion of days covered.

^b^N/A: not applicable.

^c^Neuro other: other neurological disorders.

^d^FluidsLytes: fluid and electrolyte disorders.

^e^Alcohol: alcohol abuse, referring to alcohol use disorder.

^f^Drug: drug abuse, referring to substance use disorder.

## Discussion

Our retrospective cohort study showed a change in adherence to MOUD during the COVID-19 pandemic; this change in adherence did not show up in previous aggregate and dichotomous-variable studies [13,14]. The results showed that patients’ adherence to their medication dropped between April and August 2020 and remained low until the end of 2020. Our breakdown of patients by the number of covered months showed that this was true even for patients who had relatively good levels of adherence. (As a rule of thumb, a PDC score of 0.8 has been considered as being adherent, and Warren et al [19] supported this empirically.) This association of a drop in adherence and the COVID-19 period holds when taking into account other factors that also showed a strong association. In particular, state is a significant factor, which is likely to represent a range of factors (urban or rural distribution of population, education, etc). Although these data were not available at an individual level, a machine learning–based study of adherence to MOUD found that demographic proxy data did improve models [19]; that study nevertheless still found a state effect (across the 2 states studied) even after including these data. However, differences in response to COVID-19 could also be a factor. Other factors found to be significant here—sex, age group, etc—are also broadly in line with Warren et al [19].

These results that establish an association between a drop in adherence and the COVID-19 period do not tell us the cause of the drop in adherence, such as patients ceasing to go out to get or fill medication, patients tapering themselves, or some other cause. A small-scale study (429 patients) of opioid agonist treatment use during COVID-19 in Sydney, Australia [20], found that few patients disengaged from treatment, although the social restrictions there differed from the US cohort in this study, as did the distribution of medications (66% methadone in the Australian study). Given the importance of adherence to positive treatment outcomes for opioid addiction, interventions may be warranted. These could range from simple interventions, such as sending reminders and follow-up messages, to more complex ones, such as increased frequency of scheduled follow-up visits with treatment providers and, in some instances, case management. In the context of pandemic restrictions, telehealth offers some possibilities for interventions; the Sydney study [20] found it generally adequate for most clinical reviews, and a more recent study of telemedicine in a US OUD context [21] found that for a range of indicators, telemedicine constituted a comparable alternative to in-person OUD care.

## Abbreviations

BIC: Bayesian information criterion
ICD-10: International Classification of Diseases, 10th revision
MOUD: medication for opioid use disorder
OUD: opioid use disorder
PDC: proportion of days covered

## Data Availability

Data cannot be shared publicly because the authors are not the owners of the data. Data are available from the Digital Health Cooperative Research Centre for researchers who meet the criteria for access to confidential data; access will be the same as that for the authors of this paper. Information about the process, along with a form to apply for data use and data usage agreements, are available on the web [22].
